# Addition of HER2 and CD44 to ^18^F-FDG PET–based clinico-radiomic models enhances prediction of neoadjuvant chemoradiotherapy response in esophageal cancer

**DOI:** 10.1007/s00330-020-07439-8

**Published:** 2020-11-05

**Authors:** Roelof J. Beukinga, Da Wang, Arend Karrenbeld, Willemieke P. M. Dijksterhuis, Hette Faber, Johannes G. M. Burgerhof, Véronique E. M. Mul, Riemer H. J. A. Slart, Robert P. Coppes, John Th. M. Plukker

**Affiliations:** 1 Medical Imaging Center, Department of Nuclear Medicine and Molecular Imaging, University of Groningen, University Medical Center Groningen, 9700 RB Groningen, The Netherlands; 2 Department of Surgical Oncology, University of Groningen, University Medical Center Groningen, Groningen, The Netherlands; 3 Department of Biomedical Sciences of Cells and Systems, Section Molecular Cell Biology, University of Groningen, University Medical Center Groningen, Groningen, The Netherlands; 4 Department of Pathology, University of Groningen, University Medical Center Groningen, Groningen, The Netherlands; 5 Department of Radiation Oncology, University of Groningen, University Medical Center Groningen, Groningen, The Netherlands; 6 Department of Epidemiology, University of Groningen, University Medical Center Groningen, Groningen, The Netherlands; 7 Faculty of Science and Technology, Department of Biomedical Photonic Imaging, University of Twente, Enschede, The Netherlands

**Keywords:** Positron emission tomography, Radiomics, Oncogene protein HER-2, CD44 antigen, Esophageal cancer

## Abstract

**Objectives:**

To assess the complementary value of human epidermal growth factor receptor 2 (HER2)-related biological tumor markers to clinico-radiomic models in predicting complete response to neoadjuvant chemoradiotherapy (NCRT) in esophageal cancer patients.

**Methods:**

Expression of HER2 was assessed by immunohistochemistry in pre-treatment tumor biopsies of 96 patients with locally advanced esophageal cancer. Five other potentially active HER2-related biological tumor markers in esophageal cancer were examined in a sub-analysis on 43 patients. Patients received at least four of the five cycles of chemotherapy and full radiotherapy regimen followed by esophagectomy. Three reference clinico-radiomic models based on ^18^F-FDG PET were constructed to predict pathologic response, which was categorized into complete versus incomplete (Mandard tumor regression grade 1 vs. 2–5). The complementary value of the biological tumor markers was evaluated by internal validation through bootstrapping.

**Results:**

Pathologic examination revealed 21 (22%) complete and 75 (78%) incomplete responders. HER2 and cluster of differentiation 44 (CD44), analyzed in the sub-analysis, were univariably associated with pathologic response. Incorporation of HER2 and CD44 into the reference models improved the overall performance (*R*^2^s of 0.221, 0.270, and 0.225) and discrimination AUCs of 0.759, 0.857, and 0.816. All models exhibited moderate to good calibration. The remaining studied biological tumor markers did not yield model improvement.

**Conclusions:**

Incorporation of HER2 and CD44 into clinico-radiomic prediction models improved NCRT response prediction in esophageal cancer. These biological tumor markers are promising in initial response evaluation.

**Key Points:**

*• A multimodality approach, integrating independent genomic and radiomic information, is promising to improve prediction of γpCR in patients with esophageal cancer.*

*• HER2 and CD44 are potential biological tumor markers in the initial work-up of patients with esophageal cancer.*

*• Prediction models combining *^*18*^*F-FDG PET radiomic features with HER2 and CD44 may be useful in the decision to omit surgery after neoadjuvant chemoradiotherapy in patients with esophageal cancer.*

**Supplementary Information:**

The online version contains supplementary material available at 10.1007/s00330-020-07439-8.

## Introduction

Neoadjuvant chemoradiotherapy (NCRT) followed by surgery is the preferred treatment for locally advanced (T_1_/N_1–3_/M_0_; T_2–4a_/N_0–3_/M_0_) curative-intended resectable esophageal cancer. Pathologic complete response to NCRT (γpCR) is achieved in approximately 29% of these patients [1]. For complete responders, a “wait-and-see” policy instead of esophagectomy may lead to equivalent results and avoids surgical morbidity and mortality. In evaluating tumor response, clinicians usually report features extracted from pre- and posttreatment imaging such as overall tumor volume measured on computed tomography (CT) and maximum standardized uptake value measured on ^18^F-fluorodeoxyglucose positron emission tomography (^18^F-FDG PET). However, these features have relatively unsatisfactory predictive values; and hence, no clinical relevant conclusions can be drawn from these features only [2].

These features neglect spatial information, while intratumoral heterogeneity is associated with higher levels of tumor aggressiveness and impaired response to NCRT. Spatial variation in either gene expression profiles or environmental stressors leads to multiple distinct subclonal populations with different patterns of oxygen consumption and glucose metabolism [3–5]. Intratumoral heterogeneity can be quantified using a sophisticated method called radiomics, which extracts a large number of quantitative imaging features from medical images to capture the tumor phenotype and its microenvironment. These features quantify image intensity, shape, and texture and may be of added value to traditionally clinico-pathological reports. Numerous recent studies have demonstrated that CT and/or ^18^F-FDG PET radiomics can outperform conventional radiological measurements in the prediction of γpCR in esophageal cancer patients [6–15]. Nevertheless, the use of radiomic features as quantitative image biomarkers requires further optimization and improvement in order to achieve routine clinical application.

To improve radiomics-based prediction models, incorporation of molecular targets involved in treatment resistance mechanisms is promising [16]. Studies on molecular targets are usually based on a single biopsy; therefore, they do not fully capture the spectrum of resistance clones within the individual patients’ specific tumor. Imaging features may therefore be complementary as they capture independent information from the entire tumor burden [17–19]. An important biological tumor marker which has gained predictive characteristics in esophageal cancer is human epidermal growth factor receptor 2 (HER2) [20, 21]. Activation of the HER2 proto-oncogene initiates signaling pathways leading to proliferation, inhibition of apoptosis, and tumor progression [22, 23]. Other potentially active biological tumor markers predicting resistance to NCRT are cluster of differentiation 44 (CD44) and the Hedgehog pathway markers receptor protein patched homolog 1 (PTCH1) and ligand Sonic Hedgehog (SHH) [24]. Moreover, transcription factor hypoxia-inducible factor 1-alpha (HIF1α) and the biological interaction between HER2 and CD44 may lead to upregulation of C-X-C chemokine receptor type 4 (CXCR4), which promotes tumor progression and NCRT resistance in gastroesophageal cancer [25, 26].

The aim of this study was to assess the complementary value of HER2 and its associated biological tumor markers to ^18^F-FDG PET–based clinico-radiomic prediction models to predict γpCR in esophageal cancer patients.

## Patients and methods

### Patients

**Fig. 1:**
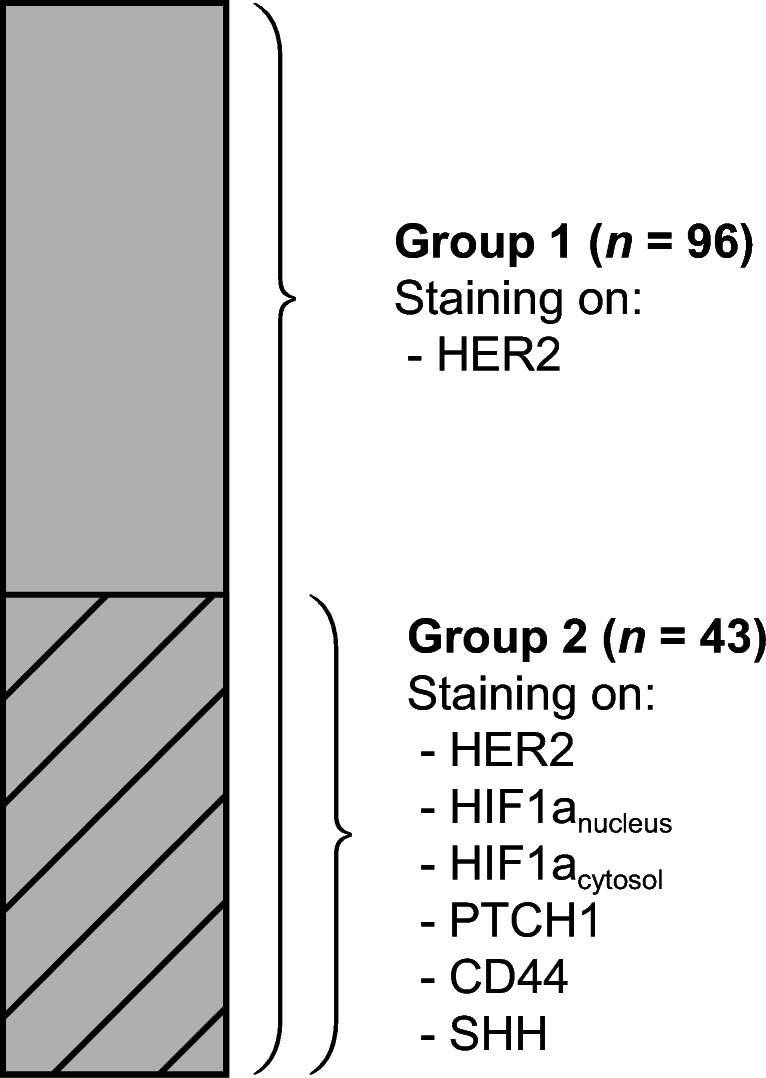
Illustration of patient groups. HER2 analysis was performed in 96 patients (group 1). In 43 of these 96 patients, sufficient pre-treatment biopsy was available to perform additional analyses (group 2)

This retrospective study was granted by the Local Institutional Review Board and obtaining informed consent was waived according to the legal regulations of our University Hospital. Patients were eligible for inclusion if they had histologically confirmed locally advanced (T_1_/N_1–3_/M_0_; T_2–4a_/N_0–3_/M_0_) esophageal cancer (according to the seventh tumor–node–metastasis classification system) and if sufficient amounts of pre-treatment biopsy material were available [27]. Moreover, patients were only included if they had a baseline ^18^F-FDG PET/CT scan to perform the radiomics analysis and received at least four of the five cycles of chemotherapy and full concomitant radiotherapy, followed by esophagectomy with curative intent in our hospital. The enrolled 96 patients were treated between March 2010 and June 2018 (group 1). In only 43 of these 96 patients (group 2) the additional required pre-treatment biopsy material was available to perform analyses of CD44, HIF1α, PTCH1, and SHH (Fig. 1).

### Treatment and pathology

NCRT was given according to the Chemoradiotherapy for Oesophageal Cancer followed by Surgery Study (CROSS) regimen, including carboplatin (AUC of 2 mg min mL^−1^) and paclitaxel (50 mg/m^2^) with concurrent radiotherapy (41.4 Gy in 23 fractions) [1]. A curative intended transthoracic open or minimal invasive esophagectomy with mediastinal and upper abdominal lymphadenectomy was performed 6–8 weeks after completion of NCRT. The primary outcome was pathologic response according to the Mandard tumor regression grade (TRG) [28]. This grading system classifies the ratio of residual vital tumor cells and the degree of NCRT-induced fibrosis and defines γpCR; γpT0N0 (Mandard TRG 1) as no residual vital tumor cells and non-γpCR (Mandard TRG 5) as no tumor regression at all.

### ^18^F-FDG PET/CT

Pre-treatment ^18^F-FDG PET/CT (Biograph mCT-64 PET/CT; Siemens) scans were acquired in radiation treatment planning position. Patients were instructed to fast except for the consumption of water for at least 6 h before scanning. Images were acquired 60 min after the intravenous injection of 3 MBq/kg ^18^F-FDG. ^18^F-FDG PET images were obtained within 2–3 min per bed position in three-dimensional setting. Images were reconstructed using a time-of-flight iterative reconstruction method (three iterations; 21 subsets) with point-spread-function correction [29]. Images were corrected for random coincidences, scatter, and attenuation (CT-based), and were smoothed with a Gaussian filter of 6.5 mm in full-width at half-maximum.

### Radiomic feature extraction

Tumor volume was delineated manually after reaching consensus between 3 collaborating researchers (RTx Workstation 1.0; Mirada Medical). In-house software was developed with Matlab 2018a (MathWorks) to process baseline ^18^F-FDG PET images and to extract 101 radiomic features [30]. Quantification of ^18^F-FDG PET offers spatial information on the rate of metabolism, which is affected by well-known risk factors for tumor NCRT resistance such as hypoxia, necrosis, and cellular proliferation. As image texture depends on image quality, the low-dose CT images were not analyzed. The extracted radiomic features consisted of 19 morphologic features (including the conventional metrics volume and total lesion glycolysis), two local intensity features, 18 statistical features (including the conventional metrics SUV_max_, SUV_peak_, and SUV_mean_), 25 gray-level co-occurrence–based features, 16 gray-level run-length–based features, 16 gray-level size-zone–based features, and five neighborhood gray-tone difference–based features. The extracted radiomic features were all listed in the IBSI reference manual and matched the IBSI benchmark values [30]. The Supplemental Method provides a more detailed description regarding the radiomic feature extraction process.

### Immunohistochemistry and scoring of biological tumor markers

Immunohistochemistry staining was performed on 5-μm tissue sections from archival biopsies using primary antibodies against HER2 (1:100, Fremont), CD44 (1:100, Biolegend), HIF1α (1:100, ABCAM), PTCH1 (1:100, ABCAM), and SHH (1:100, ABCAM). De-paraffinized tissue sections were immersed in PBS 2% hydrogen peroxidase to block endogenous peroxidase activity. Antigen retrieval was performed and the sections were incubated overnight at 4 °C with primary antibodies. Tissue sections were then incubated with biotinylated secondary antibodies at 1:300 dilutions for 1 h. The ABC complex was formed using the Vectastain Elite ABC HRP kit (Vector Laboratories). This complex was visualized with SIGMA FAST 3,3’-diaminobenzidine tablets (Sigma-Aldrich). Finally, sections were counterstained with hematoxylin and scored.

Scoring was blinded and carried out by two researchers independently. Discordant cases and random slides of each marker were validated by a pathologist specializing in upper gastrointestinal cancer. If at least 5 clustered tumor cells were stained, HER2 was scored according to standardized methods in a discrete scale of 0, 1+, 2+, and 3+ [22, 31]. Following the guidelines in the immunohistochemical evaluation of HER2, this scale was dichotomized. Tumors with score 0 were considered negative while tumors with score 3+ were considered positive. As tumors with 1+ represent the majority of false-negative results and tumors with 2+ represent the majority of false-positive results, these cases were subjected to fluorescence in situ hybridization to confirm the HER2 status [22, 23, 32]. HIF1α was scored separately as it is expressed either in the cytosol (normoxia) or in the nucleus (hypoxia). CD44, HIF1α_cytosol_, and PTCH1 were scored using the 15-point immuno-reactivity score as described in the Supplemental Methods. HIF1α_nucleus_ and SHH intensity were categorized as either present or absent [33].

### Statistical analysis

Statistical analysis was performed with R 3.5.3 open-source software using the regression modeling strategies package (version 5.1-3), available from the Comprehensive R Archive Network (http://www.r-project.org). The outcome variable, categorized as γpCR versus non-γpCR (Mandard TRG 1 vs. 2–5), was modeled using logistic regression. Biological tumor markers with a likelihood-ratio test (LRT) *p* value < 0.2 in univariable logistic regression analysis were preselected and entered separately to three reference models. The reference models were constructed based on clinical features as listed in Table 1 (model 1), radiomic features (model 2), and both clinical and radiomic features (model 3). Prior to the final selection of radiomic features, the feature space was reduced by agglomerative hierarchical clustering with average linkage to group both radiomic features and patients in clusters based on their Spearman rank correlation coefficient. Clusters were formed when groups of nodes in the dendrogram were < 60% of the maximum linkage. From each feature cluster, a representative radiomic feature was selected based on the lowest univariable LRT *p* value per cluster. Only representative radiomic features with LRT *p* < 0.2 were subjected to the final feature selection process based on least absolute shrinkage and selection operator, a technique for L1-norm regularization.

**Table 1:** Patient and tumor characteristics

	Group 1		Group 2	
Characteristics	γpCR (*n* = 21)	Non-γpCR (*n* = 75)	γpCR (*n* = 9)	Non-γpCR (*n* = 34)
Sex				
Male	14 (18%)	64 (82%)	7 (19%)	29 (81%)
Female	7 (39%)	11 (61%)	2 (29%)	5 (71%)
Age (median (IQR)) (years)	65 (7)	63 (10)	65 (6)	63 (10)
Histology				
Adenocarcinoma	16 (18%)	72 (82%)	8 (%)	34 (81%)
Squamous cell carcinoma	5 (62%)	3 (38%)	1 (100%)	0 (0%)
Tumor location				
Distal esophagus/GEJ	21 (22%)	75 (78%)	9 (21%)	34 (79%)
Tumor length (median (IQR)) (cm)	5.0 (5.3)	6.0 (4.0)	3.0 (6.5)	6.0 (4.0)
Clinical T-stage				
T1 and T2	6 (55%)	5 (45%)	3 (50%)	3 (50%)
T3 and T4a	15 (18%)	70 (82%)	6 (16%)	31 (84%)
Clinical N-stage				
N0 and N1	19 (28%)	49 (72%)	8 (27%)	22 (73%)
N2 and N3	2 (7%)	26 (93%)	1 (8%)	12 (92%)
Number of chemotherapy cycles				
4	4 (22%)	14 (78%)	2 (20%)	8 (80%)
5	17 (22%)	61 (78%)	7 (21%)	26 (79%)
Mandard tumor regression grade				
1	21 (100%)	0 (0%)	9 (100%)	0 (0%)
2	0 (0%)	24 (100%)	0 (0%)	12 (100%)
3	0 (0%)	33 (100%)	0 (0%)	17 (100%)
4	0 (0%)	16 (100%)	0 (0%)	4 (100%)
5	0 (0%)	2 (100%)	0 (0%)	1 (100%)

*Abbreviations*: *γpCR*, pathologic complete response; *IQR*, interquartile range; *GEJ*, gastroesophageal junction

The performance of the constructed models was assessed using overall performance, discrimination, and calibration measures. Discrimination describes the ability to discriminate between γpCR and non-γpCR and was measured using the AUC and the discrimination slope. Calibration refers to the agreement between observed outcomes and predictions, and was evaluated with calibration plots. Measures of overall performance are composed of discrimination and calibration characteristics of the model and include the Nagelkerke R^2^ and the Brier score [34]. All measures were corrected for model optimism by internal validation with bootstrapping (20,000 repetitions).

## Results

### Patient characteristics

Table 1 displays the patient and tumor characteristics. Seventy-eight patients (81%) in group 1 and 33 patients (77%) in group 2 received all 5 cycles of chemotherapy, while 18 patients (19%) in group 1 and 10 patients (23%) in group 2 received 4 cycles of chemotherapy. In group 1, 21 patients (22%) and 75 patients (78%) were scored as γpCR and non-γpCR, compared to respectively 9 patients (21%) and 34 patients (79%) in group 2.

### Immunohistochemistry scores

HER2 amplification could not be established in two patients in group 1 and one patient in group 2 and was therefore scored as missing. Immunohistochemical staining for all remaining samples was successfully performed. The distributions of the immunohistochemical scores of the six analyzed biological tumor markers are provided in Table 2. HER2 and CD44 were the only significant biological tumor markers at univariable logistic regression analysis (LRT *p* value = 0.043 and LRT *p* value = 0.051) and were therefore considered for further analysis. HIF1α_nucleus_, HIF1α_cytosol_, PTCH1, and SHH were not found to be significant (LRT *p* values of 0.847, 0.367, 0.443, and 0.236 respectively).

**Table 2:** The distribution of the immunohistochemistry scores

	Group 1		Group 2	
Tumor markers	γpCR (*n* = 21)	Non-γpCR (*n* = 75)	γpCR (*n* = 9)	Non-γpCR (*n* = 34)
HER2				
Negative	19 (90%)	57 (76%)	8 (89%)	26 (76%)
Positive	1 (5%)	17 (23%)	0 (0%)	8 (24%)
Missing	1 (5%)	1 (1%)	1 (11%)	0 (0%)
HIF1α_nucleus_				
Negative			8 (89%)	31 (91%)
Positive			1 (11%)	3 (9%)
HIF1α_cytosol_				
Negative			2 (22%)	13 (38%)
Positive			7 (78%)	21 (62%)
PTCH1				
Negative			2 (22%)	4 (12%)
Positive			7 (78%)	30 (88%)
CD44				
Negative			1 (11%)	15 (44%)
Positive			8 (89%)	19 (56%)
SHH				
Negative			0 (0%)	3 (9%)
Positive			9 (100%)	31 (91%)

*Abbreviations*: *γpCR*, pathologic complete response; *HER2*, human epidermal growth factor receptor 2; *CD44*, cluster of differentiation 44; *HIF1α*, hypoxia-inducible factor alpha; *PTCH1*, protein patched homolog 1; *SHH*, Sonic Hedgehog

High HER2 expression was found to be more prevalent in impaired responders. To be more specific, 17 of the 18 HER2-positive patients developed non-γpCR (negative predictive value of 94%) and HER2 was negative in 19 of the 20 patients with γpCR (sensitivity of 95%). Moreover, 18 of the 18 (100%) HER2-positive patients had a clinical T_3–4a_ tumor and 11 of the 76 (14%) HER2-negative patients had a clinical T_1–2_ tumor (*p* value = 0.12, Fisher’s exact test). Supplemental Figure 1 demonstrates representative pictures of low (0) and high (3+) HER2 expression patterns. CD44 amplification was found more frequently in complete responders than in incomplete responders. From 16 CD44-negative patients, 15 developed a non-γpCR (positive predictive value of 94%), while 8 of the 9 patients with γpCR were CD44-positive (sensitivity of 89%). Only 1 of the 16 (6%) CD44-negative patients had a T_1–2_ tumor, while 22 of the 27 (81%) CD44-positive patients had a T_3–4a_ tumor (*p* value = 0.35, Fisher’s exact test). In group 2, all 6 patients who were both HER2-positive and CD44-positive developed a non-γpCR.

### Reference model construction

**Fig. 2:**
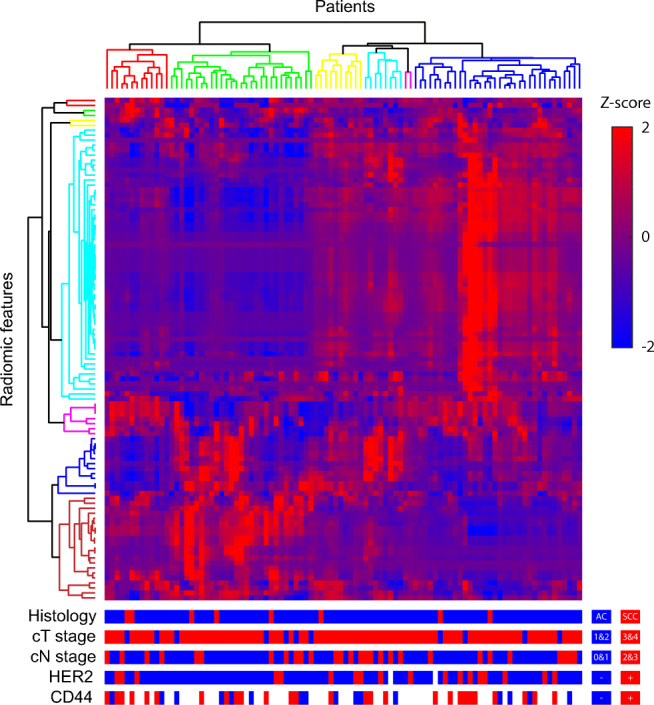
Heatmap for radiomic feature expression with a *Z*-score. Hierarchical clustering revealed 7 radiomic feature clusters (different tree colors along the y-axis) and 6 patient clusters (different tree colors along the x-axis) with similar radiomic feature expression patterns. Representative radiomic features corresponding to the feature clusters (from top to bottom in the tree) were inverse variance, coarseness, Moran’s I index, second measure of information correlation, elongation, Geary’s C measure, and long-run low gray-level emphasis. We tested whether clinico-pathological features (histology, clinical T-stage, and clinical N-stage) and biological expression (HER2 and CD44) are distributed equally across different patient clusters. The fact that no association was found suggests independent information of these multimodal features

Hierarchical clustering revealed 7 groups of correlated features (Fig. 2). The representative features corresponding to these feature clusters (in Fig. 2, from top to bottom in the tree) were inverse variance, coarseness, Moran’s I index, second measure of information correlation, elongation, Geary’s C measure, and long-run low gray-level emphasis. The conventional metrics (volume, total lesion glycolysis, SUV_max_, SUV_peak_, and SUV_mean_) were not selected as representative features. Model 1 was constructed based on histology and clinical T-stage, while Geary’s C measure and long-run low gray-level emphasis were selected from the representative features for model 2. Geary’s C measure is an indicator of spatial autocorrelation for finding repeating metabolic patterns. Long-run low gray-level emphasis is dependent on long sets of aligned voxels with low metabolic activity. Model 3 was the full model incorporating all variables of models 1 and 2.

### Complementary value of the biological tumor markers

**Fig. 3:**
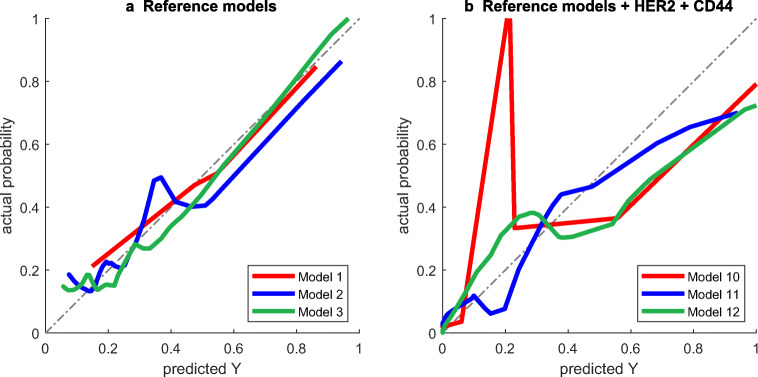
Calibration plots of reference prediction models 1, 2, and 3, without (**a**) and with (**b**) HER2 and CD44 incorporated

As no associations were found between the patient clusters and histology, clinical T-stage, clinical N-stage, HER2, and CD44 (*χ*^2^ test; Fig. 2), we assumed that there was no connection between gene expression profiles and imaging phenotypes. HER2 and CD44 were separately added to the abovementioned reference models to evaluate their complementary value. The performance measures of all constructed models are depicted in Table 3. The reference models had the following performance measures: overall model performance (*R*^2^_M1_ = 0.140, *R*^2^_M2_ = 0.103, and *R*^2^_M3_ = 0.173), discrimination (AUC_M1_ = 0.657, AUC_M2_ = 0.654, and AUC_M3_ = 0.685), and calibration (Intercept_M1_ = 0.086 and Slope_M1_ = 0.817; Intercept_M2_ = 0.047 and Slope_M2_ = 0.826; and Intercept_M3_ = 0.035 and Slope_M3_ = 0.895). Although separate incorporation of HER2 or CD44 into the reference models did not improve model performance, incorporating HER2 and CD44 simultaneously yielded substantially improved overall performance (*R*^2^_M10_ = 0.221, *R*^2^_M11_ = 0.270, and *R*^2^_M12_ = 0.225) and discrimination (AUC_M10_ = 0.759, AUC_M11_ = 0.857, and AUC_M12_ = 0.816). However, the calibration plots (Fig. 3) show that model 10 underestimated the probability of achieving a γpCR in low-risk patients, while models 10–12 slightly overestimated the probability in high-risk patients.

**Table 3:** Performance of prediction models with and without biological tumor markers

Model	AIC	R^2^	Brier	AUC	DS	Int	Slope
M1	94.7	0.140	0.158	0.657	0.083	0.086	0.817
M2	97.4	0.103	0.163	0.654	0.058	0.047	0.826
M3	92.3	0.173	0.151	0.685	0.105	0.035	0.895
M4 = M1 + HER2	90.9	0.133	0.153	0.700	0.075	0.041	0.894
M5 = M2 + HER2	92.3	0.115	0.155	0.694	0.063	0.033	0.880
M6 = M3 + HER2	89.1	0.162	0.147	0.700	0.094	0.030	0.894
M7 = M1 + CD44	45.1	0.127	0.163	0.739	0.046	0.072	0.758
M8 = M2 + CD44	47.3	0.087	0.174	0.748	0.017	0.064	0.701
M9 = M3 + CD44	46.5	0.114	0.175	0.737	0.016	0.080	0.643
M10 = M1 + HER2 + CD44	40.5	0.221	0.146	0.759	0.106	0.069	0.763
M11 = M2 + HER2 + CD44	41.2	0.270	0.135	0.857	0.134	0.036	0.834
M12 = M3 + HER2 + CD44	42.0	0.225	0.150	0.816	0.073	0.060	0.724

*Abbreviations*: *M1*, clinical reference model; *M2*, radiomic reference model; *M3*, clinico-radiomic reference model; *HER2*, human epidermal growth factor receptor 2; *CD44*, cluster of differentiation 44; *AIC*, Akaike Information Criterion; *R*^*2*^, Nagelkerke R^2^; *Brier*, Brier score; *AUC*, area under the receiver operating characteristic; *DS*, discrimination slope; *Int*, intercept

## Discussion

The value of this study is the combination of biological tumor markers from pre-treatment tumor biopsies and intratumoral ^18^F-FDG spatial distribution capturing information from the entire tumor burden. The improvement of the reference prediction models by the simultaneous addition of HER2 and CD44 demonstrates the value of this concept. Prediction model 11, composed of ^18^F-FDG PET–based radiomic features (Geary’s C measure and long-run low gray-level emphasis) and biological tumor markers (HER2 and CD44), was the preferred prediction model as it showed the highest observed level of overall performance and discrimination. The model had good ability to differentiate between γpCR and non-γpCR, but in some cases it was less useful to predict the actual probability of γpCR due to its fairly well calibration.

We found that HER2 activation was associated with a lower probability of achieving a γpCR. This is consistent with studies which showed the association between overexpression of HER2 with poor survival and therapy response [17, 20, 23]. Another study showed that enrichment of postoperative HER2 levels in surgical resection material compared to pre-treatment biopsies was associated with poor pathologic response [35]. In our study, immunohistochemical activation of CD44 was related to a higher probability of achieving γpCR. CD44 is a cell-surface transmembrane glycoprotein that has been observed to be present in cancer stem cells of esophageal cancer, a subpopulation of cells with the capacity of self-renewal. In vitro, the combination of CD44^+^/CD24^−^ subpopulation of esophageal cancer cells has shown to be more resistant to NCRT [36]. Although the reference prediction models showed similar performance when HER2 and CD44 were added separately, adding both variables simultaneously yielded improved performances. This might be explained by the biological interaction between HER2 and CD44, which leads to upregulation of CXCR4 in gastroesophageal cancer and may enhance NCRT resistance [25, 26]. Contradictory results have been reported regarding the association of HER2 and CD44 expression and T-stage [37, 38]. In this study, we did not observe any association; and therefore, these variables may contribute to the prediction model as independent variables. This was confirmed by the fact that model performance improved when HER2 (model 4) and CD44 (model 7) were separately added to the clinical reference model (model 1), which was constructed based on histology and clinical T-stage. According to the general guidelines, HER2 status should only be verified in patients with advanced esophageal adenocarcinoma who are potential candidates for HER2-targeting [22]. HER2 and CD44 status assessment is currently not part of the pre-treatment staging. However, the findings of this study supported by literature suggest a potential value of adding pre-treatment HER2 and CD44 status in NCRT response prediction in locally advanced esophageal cancer.

Although adding HER2 and CD44 complements our reference prediction models, it should be noted that some of the constructed models were based on a relatively small number of patients (*n* = 43), which increases the risk of model overfitting. The reference prediction models were slightly better calibrated than the preferred prediction model, but had substantially lower overall performance and discrimination. However, well-calibrated models which have poor discrimination do not have any clinical value. Vice versa, the usability of the model is only limited if the model is poorly calibrated at the clinically chosen threshold to omit surgery. To prevent optimistic performance estimates, all measures were corrected for optimism by internal validation. However, as even internal validation may not be indicative of the final model’s performance in future settings, the model should be validated in an independent validation cohort. Moreover, the reproducibility of immunohistochemical evaluation and radiomic feature extraction should be established before it can be safely implemented and generalized in a clinical setting. Immunohistochemical protocols may differ among institutes and results depend on the level of experience of the researcher. In this study, inter-rater variation was minimized by the independent evaluation of two researchers. Discordant cases were validated by an experienced pathologist. Moreover, only a few studies have reported on measurement errors (i.e., reliability, reproducibility, or repeatability) of radiomics [39–41]. Although further research is warranted, initial results show that most radiomic features are sensitive to numerous confounding factors including image acquisition, reconstruction protocols, or delineation methods. Standardization in the radiomics extraction workflow therefore remains essential to achieve routine clinical adoption.

This study indicates that HER2 and CD44 in the initial work-up could be useful biological tumor markers in predicting γpCR to NCRT in esophageal cancer. As genomic and ^18^F-FDG PET–based radiomic features may yield independent complementary information, integration of these multimodal features may improve the performance of prediction models.

## Supplementary Information

ESM 1 (DOCX 4820 kb)

## Abbreviations

^18^F-FDG PET: ^18^F-Fluorodeoxyglucose positron emission tomography
CD44: Cluster of differentiation 44
CROSS: Chemoradiotherapy for Oesophageal Cancer followed by Surgery Study
CT: Computed tomography
CXCR4: C-X-C chemokine receptor type 4
HER2: Human epidermal growth factor receptor 2
HIF1α: Hypoxia-inducible factor 1-alpha
LRT: Likelihood-ratio test
NCRT: Neoadjuvant chemoradiotherapy
PTCH1: Protein patched homolog 1
SHH: Sonic Hedgehog
TRG: Tumor regression grade
