# Supplementary material for: Addition of HER2 and CD44 to 18F-FDG PET–based clinico-radiomic models enhances prediction of neoadjuvant chemoradiotherapy response in esophageal cancer
Source: Eur Radiol. 2020 Nov 5;31(5):3306–14. doi: 10.1007/s00330-020-07439-8 (PMC8043921; doi:10.1007/s00330-020-07439-8)
Supplement: Supplementary file 1 — (DOCX 4820 kb) [file 330_2020_7439_MOESM1_ESM.docx]

**Electronic Supplementary Material**

**Supplemental Figures**


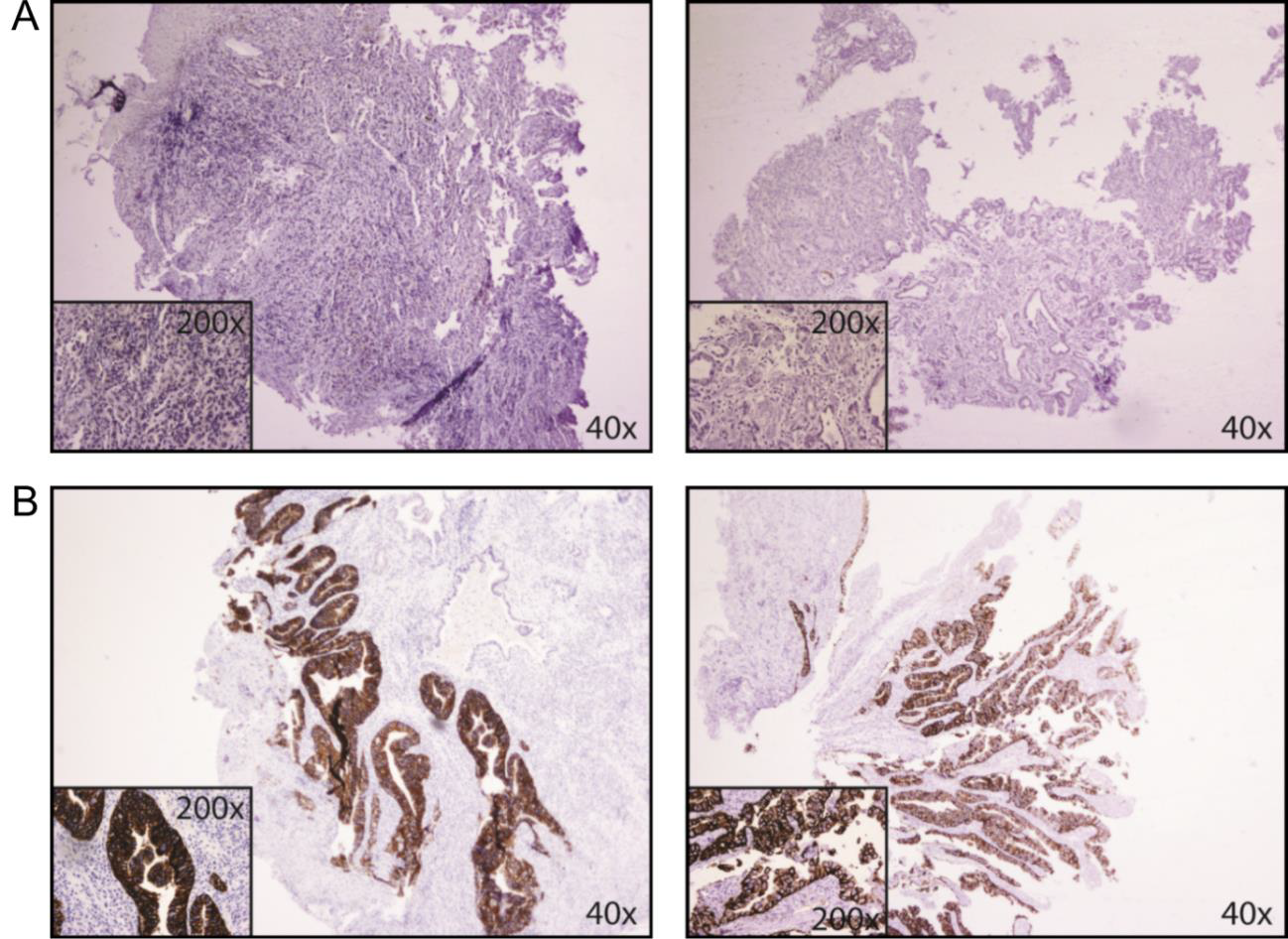


Supplemental Figure 1. Representative immunohistochemistry staining of 2 patients who were scored as immunohistochemistry 0 (*A*) and 2 patients who were scored as immunohistochemistry 3+ (*B*) for HER2 expression.

**Supplemental Methods**

***Radiomic feature extraction***

In-house software was developed with Matlab 2018a (Mathworks, Natick, Mass) to process ^18^F-FDG PET images and to extract 101 radiomic features. SUV was used as a relative measure of ^18^F-FDG uptake and was corrected for serum glucose level [1].

Original voxel dimensions were up-sampled from 3.2×3.2×2.0 to 2.0×2.0×2.0 mm isotropic voxel-dimensions using trilinear spline interpolation. The interpolation mesh grid was positioned at the center of the original grid. Voxels enclosed for ≥50% coverage were included. The extracted radiomic features consisted of 19 morphologic features, two local intensity features, 18 statistical features, 25 gray-level co-occurrence–based features, 16 gray-level run-length–based features, 16 gray-level size-zone–based features, and five neighborhood gray-tone difference–based features. The extracted radiomic features matched the IBSI benchmark values [2]. Textural features were extracted from discretized image stacks to reduce the continuous-scaled SUV to a limited number of gray levels and to reduce image noise. Voxels were discretized in 0.5 g/mL increments starting at 0 g/mL. Images were analyzed in three dimensions with a connectivity of 26 voxels (13 angular directions and a Chebyshev distance of one).

Gray-level co-occurrence–based and gray-level run-length–based features were computed from a single matrix aggregating all three-dimensional directional matrices. All radiomic features were normalized into the range [0,1].

***Immuno-reactivity score (IRS)***

CD44, HIF1αcytosol, and PTCH1 were scored using the fifteen-point immuno-reactivity score (IRS), calculated by multiplying the percentage of positive cells by the stain intensity score [3]. The percentage of positive cells was categorized into six levels: level

0 = 0%, 0% < level 1 < 5%, 5% ≤ level 2 < 25%, 25% ≤ level 3 < 50%, 50% ≤ level 4 < 75%, and 75% ≤ level 5 ≤ 100%. Stain intensity was categorized into 4 levels by labeling negative as 0, weak as 1, medium as 2, and strong as 3 [3]. A dichotomous immunoscore was created by labeling absent/low expression (IRS 0-5) as 0 and moderate/high expression (IRS 6-15) as 1 [3]. SHH demonstrated the maximum percentage of positive cells (≥75% to 100%) in the majority of the cases. Therefore, SHH intensity was only scored dichotomously by categorizing stain intensity into absent/low expression (0-1 intensity) and moderate/high expression (2-3 intensity) [3].

**Supplemental References**

[1] Boellaard R, Delgado-Bolton R, Oyen WJG, et al. FDG PET/CT: EANM procedure guidelines for tumour imaging: Version 2.0. Eur J Nucl Med Mol Imaging. 2015;42:328-354.

[2] Zwanenburg A, Leger S, Vallières M, et al. Image biomarker standardisation initiative - feature definitions. CoRR 2016; abs/1612.07003.

[3] Honing J, Pavlov KV, Mul VE, et al. CD44, SHH and SOX2 as novel biomarkers in esophageal cancer patients treated with neoadjuvant chemoradiotherapy. Radiother Oncol. 2015;117:152-158.
